# Corrosion Assessment in Reinforced Concrete Structures by Means of Embedded Sensors and Multivariate Analysis—Part 1: Laboratory Validation

**DOI:** 10.3390/s23218869

**Published:** 2023-10-31

**Authors:** José Enrique Ramón-Zamora, Josep Ramon Lliso-Ferrando, Ana Martínez-Ibernón, José Manuel Gandía-Romero

**Affiliations:** 1 Instituto de Ciencias de la Construcción Eduardo Torroja, CSIC, c/Serrano Galvache, 4, 28002 Madrid, Spain; jose.ramon@ietcc.csic.es; 2 Research Institute for Molecular Recognition and Technological Development (IDM), Universitat Politècnica de València, Camino de Vera, s/n., 46022 Valencia, Spain; anmarib@arqt.upv.es (A.M.-I.); joganro@csa.upv.es (J.M.G.-R.); 3 Department of Architectural Constructions, School of Architecture, Universitat Politècnica de València, Camino de Vera, s/n., 46022 Valencia, Spain

**Keywords:** reinforced concrete structures, corrosion, structural health monitoring, structural management, sensors, corrosion monitoring, multivariate analysis

## Abstract

Reinforced Concrete Structures (RCS) are a fundamental part of a country’s civil infrastructure. However, RCSs are often affected by rebar corrosion, which poses a major problem because it reduces their service life. The traditionally used inspection and management methods applied to RCSs are poorly operative. Structural Health Monitoring and Management (SHMM) by means of embedded sensors to analyse corrosion in RCSs is an emerging alternative, but one that still involves different challenges. Examples of SHMM include INESSCOM (Integrated Sensor Network for Smart Corrosion Monitoring), a tool that has already been implemented in different real-life cases. Nevertheless, work continues to upgrade it. To do so, the authors of this work consider implementing a new measurement procedure to identify the triggering agent of the corrosion process by analysing the double-layer capacitance of the sensors’ responses. This study was carried out on reinforced concrete specimens exposed for 18 months to different atmospheres. The results demonstrate the proposed measurement protocol and the multivariate analysis can differentiate the factor that triggers corrosion (chlorides or carbonation), even when the corrosion kinetics are similar. Data were validated by principal component analysis (PCA) and by the visual inspection of samples and rebars at the end of the study.

## 1. Introduction

Civil engineering structures play an extremely important role in a country’s socio-economic activities [1]. In addition, these infrastructures are generally the most expensive national investment and an asset of any country [2]. In most cases, reinforced concrete is chosen to build them [3] for different reasons: easily obtainable raw materials and a relatively simple manufacturing process [4]. In addition, its application covers a wide variety of works thanks to its versatility, workability, properties, and aesthetic possibilities, which confer it a very high potential [5,6,7]. Another characteristic of reinforced concrete that distinguishes it from other building materials is durability, which is defined as its ability to resist any deterioration process to maintain its original form, quality, and serviceability [8]. Yet this material is not as durable as once thought [9].

Since the 1990s, one of the main causes to be detected to affect reinforced concrete structures (RCS) durability is rebar corrosion [10,11,12,13,14]. This means that the end of the lifespan of many civil infrastructures is prematurely reached [15,16]. Hence, the deterioration analysis of concrete infrastructure and the service life predictions of these constructions are some of the greatest civil engineering challenges that the developed world faces [17,18].

On embedded steel corrosion, it is well known that a passive layer initially appears on rebars thanks to the high alkalinity of the cementitious matrix, which allows corrosion to be initially considered negligible [19,20]. However, some situations, such as chlorides reaching metal or concrete carbonation, induce corrosion in an active state in RCSs [21,22]. Knowing the rebar corrosion level is fundamental to avoiding damage, costly repair work, or, in some extreme cases, even fatalities.

This situation has resulted in an obligation to set up protocols to evaluate RCSs. For example, both the Spanish Structural Concrete Instruction (EHE-08) and the current Structural Code (the new Spanish Standard to regulate concrete, steel, and mixed concrete/steel structures for building and civil engineering purposes) [23,24] indicate that project designers must devise a maintenance strategy along with the building project. Both documents indicate that evaluation and maintenance protocols must be defined by project designers and carried out by the property developer throughout the structure’s service life. These stakeholders are not completely familiar with the particular problems of RCSs and, more specifically, with the corrosion process of embedded rebars.

Visual inspection methods are still a tool that predominates in the monitoring and control programmes of these constructions [25], although previous works have cited their limitations [26]. This is a complex task that is restricted to detecting damage to parts’ exteriors and depends on the subjectivity of the worker who makes the inspection [27,28]. It is also a costly and time-consuming methodology that often involves staff displacements and having to stop the structure being used, which makes its operation poor [26]. We must also bear in mind that it is limited to accessible structural elements and ignores having to analyse buried or immersed elements, which cannot be studied. Another disadvantage of visual inspection methods is later corrosion process detection. The corrosion phenomenon becomes visible on structural elements’ exteriors once corrosion damage is quite advanced when rust stains or cracks can be seen [29,30]. Therefore, it reduces the reliability of the results obtained by this evaluation protocol.

To face these drawbacks, on-site measurement (OSM) tools are one of the most widely used alternatives and can be classified into two main groups: qualitative and quantitative. In qualitative terms, measurements tend to be taken to study the cover layer’s resistivity [31,32,33] and to supplement the information that an analysis of rebars’ corrosion potential contributes [34,35,36]. Other authors have proposed using more sophisticated tools to measure the cover layer’s permeability [37] or to detect faults in concrete by means of electromagnetic radar or GPR (ground-penetrating radar) [38,39]. However, all these tests only serve to determine the risk of corrosion existing on rebars. Corrosion rate measurements are virtually the sole viable means of assessing rebar corrosion without removing the concrete cover [40]. To analyse it, several tools based on the guard ring and galvanostatic pulse exist [41,42,43,44,45,46,47,48,49,50]. Some of them are able to analyse corrosion with an analysis of cover layer resistivity, even with no electrical connection with rebars but simply supporting the measurement equipment on the surface [51]. Such equipment is used to analyse the RCS’ corrosion on-site and to ensure good reliability. However, they are expensive and must be managed by qualified personnel, who must travel to the structure to conduct the study. For structures protected with cover layers or paint, these protective measures must be removed, which implies aesthetic damage. Moreover, such equipment cannot be used to analyse inaccessible elements, such as buried or submerged pieces.

**Figure 1:**
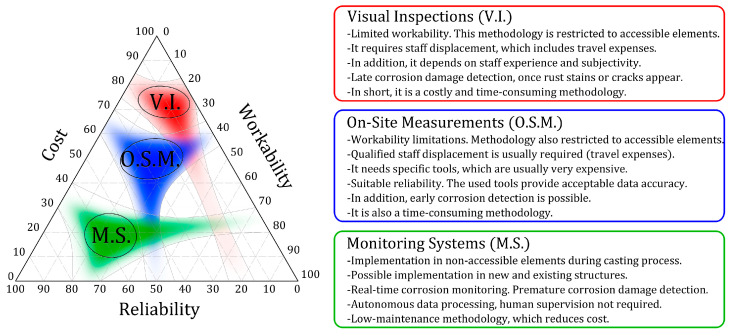
Structural Health Monitoring of Reinforced Concrete Structures, different approaches: advantages and disadvantages.

All these aspects are crucial because it is not easy to establish protocols for periodic inspections owing to the cost and drawbacks associated with staff displacements and accessibility conditions. These limitations require embedded monitoring systems for structure evaluation and management to be developed and implemented [25]. In the RCS corrosion area, Structural Health Monitoring and Management (SHMM) using embedded sensors has progressed to increase safety and provide cost-effective maintenance programs for new and existing RCSs [25]. Another advantage of these systems is premature corrosion detection, which facilitates immediate intervention. Figure 1 shows the comparative outline of the different SHMM strategies in the RCS corrosion field.

Many authors have proposed qualitative sensors to monitor parameters related to corrosion in RCSs, for instance, fibre optics [52,53] or fibre bragg grating sensors [54]. Nonetheless, systems that provide a numerical value of rebar corrosion are required, that is, quantitative tools. Some examples of such are those presented by Andrade and Martínez [55] and Pereira et al. [56]. They are based on small 3-electrode cells embedded in concrete. Xu et al. [57] proposed a similar example, but one with cells found on different concrete covers, to study how chlorides or carbonation can advance. Authors like Broomfield et al. [58] opted for a design that includes bigger elements mounted onto a frame, which is fitted to the rebar cage. Karthick et al. [59] proposed embedding only the working electrode in concrete, while other measurement cell elements (counter electrode and reference electrode) remain on the mounted surface. Duffó and Farina [60] designed a small-sized multisensor to analyse other parameters like temperature, oxygen availability, the presence of chlorides, or concrete resistivity in parallel with the corrosion rate study. This approach has also been applied by other authors who have proposed their own models [61,62]. Attempts have been made to detect carbonation advances or chloride penetration by means of potentiometric [63,64] or galvanic sensors [65,66]. However, their result interpretation is not easy because minor changes in humidity or temperature significantly affect the taken measurements. Besides, these sensors’ stability has been studied only for relatively short periods [67].

Despite all the existing examples, implementing embedded corrosion sensors in real structures is still quite scarce. This is because embedded monitoring systems for corrosion analysis in RCSs still pose some challenges to being considered a tool of generalised use that can replace conventional visual inspections and on-site corrosion measurement techniques. These challenges can be summarised as follows:

- Implementing sensor networks in real-world structures is still a difficult task. The aforementioned examples have been individually validated by laboratory testing. Nevertheless, as monitoring a structure requires many control points to analyse different zones, it is necessary to generate sensor networks that work in a coordinated manner rather than isolated elements;
- These systems must be autonomous. This involves having to implement central units that manage the data collected by the sensors distributed all over the structure and are capable of processing it to offer clear data directly and automatically. As other authors have pointed out, this includes data transfer, processing, plotting, and even websites to store data and present results [1,68]. This requirement also implies making hardware and system communication investments;
- The stakeholders who intervene in developing and building civil infrastructure still need to be made aware of the importance of embedded monitoring systems. More often than not, there are still no concerns at all about structure maintenance, and standards do not significantly highlight the importance of structure management;
- The method to set up systems in real work and its integration with the other tasks to be performed while building works. Embedded monitoring systems must be set up simply and quickly with no maintenance requirements to considerably lower their cost. For this purpose, the durability and robustness of the employed components (durability longer than the structure’s foreseeable service life) must also be taken into account. Some authors point out that the main constraints of these systems are still linked to sensors’ durability and stability over time [69];
- The economic aspect. It is fundamental to be competitive compared with traditional evaluation and management systems, such as visual inspection or on-site corrosion measurements.

One of the examples that has attempted to respond to these challenges is the INESSCOM (Integrated Sensor Network for Smart Corrosion Monitoring) monitoring system. This tool appears in previous works [70,71,72]. It is a smart system composed of control points located in different areas of the analysed structure. A corrosion sensor is embedded at each control point. Sensors are made using steel with the same properties as embedded rebars, which reduces their cost. In addition, an innovative measurement method for corrosion analysis is used. It is defined as Pulse Step Voltammetry (PSV). This technique has been previously validated [71,73,74,75,76]. The advantages that distinguish the PSV method from other techniques are that it allows work to be done with 2-electrode cells and that its processing can be automated. Thanks to these advantages, this tool has already been implemented in real structures and works autonomously [70], although the authors are still working on upgrading and refining it [77,78].

Therefore, this study aims to update the INESSCOM system so that it is able to not only measure rebar corrosion intensity but can also autonomously determine the triggering agent of corrosion by studying complementary parameters, in this case, the sensor’s electric double-layer capacitance. To do so, it proposes implementing a new measurement procedure on the sensors based on the voltammogram study (∆I−∆E) obtained after applying cyclic sweep voltammetry (CSV). This implies making a major contribution to the INESSCOM system and SHMM methods for representing a new improvement that centres on these tools’ autonomous work. The system will not only be able to detect rebar corrosion but will also provide information about the factors that have triggered the process. This will be key for RCSs evaluation and management to detect corrosion early, and it will help to determine the most suitable type of repair needed to mitigate such processes in the future. To do so, this work presents an experimental plan to analyse the corrosion of 27 concrete samples in which corrosion sensors like those employed by INESSCOM are embedded. These samples were previously exposed to different atmospheres for 18 months. The corrosion intensity analysis and sensors’ double-layer capacitance allowed the triggering agent of corrosion processes to be autonomously identified, even when the recorded corrosion kinetics were similar. This study was validated with different corrosion intensity measurement techniques, by a principal component analysis (PCA) of the obtained results, and by the visual inspection of samples and sensors when finishing the experimental plan.

## 2. Materials and Methods

### 2.1. Test Specimens

**Figure 2:**
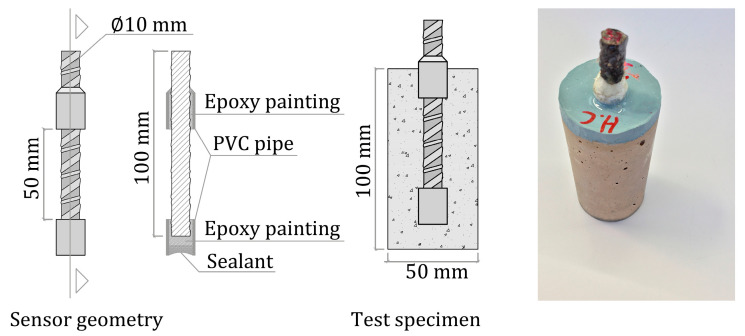
Test specimen description.

As depicted in Figure 2, 27 cylindrical specimens (Ø50 mm × 100 mm height) were manufactured. On the central axis of each sample, one sensor was embedded (steel B 500 SD), whose surface in contact with concrete was limited to 50 linear mm (1571 mm^2^). To go about this, the sensor was partially protected with PVC piping and filled with epoxy paint. The top part was left uncovered to make an electric connection, but it was protected with Vaseline to prevent corrosion. The upper sample face of the test specimens was also protected with epoxy paint. The sensors’ concrete cover layer measured 20 mm.

### 2.2. Materials

To manufacture samples, micro-concrete with a w/c ratio of 0.8 was employed. The applied concrete mixture is shown in Table 1. This mix goes beyond the standard limits for structural concrete but was designed to achieve a high porosity degree, accelerate the diffusion of aggressive agents, and obtain high corrosion levels in a short time. The employed cement was CEM I 52.5 R/SR.

### 2.3. Exposure Conditions

After the casting process, samples were stored in a curing chamber (20 ± 2 °C with a relative humidity (RH) higher than 95%) until they reached the age of 28 days. The specimens were then divided into three groups (A, B, C) with nine samples each. Group A was subjected to accelerated carbonation. Based on the phenolphthalein tests and the weight control carried out on other similar samples, it took 42 days to completely carbonate the test specimens. Furthermore, groups A and B were immersed in a 35 g/L NaCl solution (pH ≈ 7), while Group C was placed in a saturated ${Ca(OH)}_{2}$ solution (pH ≈ 13). Specimens were left under these conditions for 18 months, and subsequently, electrochemical tests were conducted. To obtain the variation coefficients of the different measurement methods employed, each specimen was tested five times by distinct techniques, as later described.

### 2.4. Testing Procedure

The testing processes undertaken in this stage were:

- First, the corrosion potentials ($E_{CORR}$) of sensors were measured by a high-impedance voltmeter (multimeter Keithley 2000) using a calomel reference electrode (SCE of Radiometer Analytical XR110) following Standard ASTM C876 [79]. The reference electrode was partially immersed in the exposure solution, depending on each group. The value was recorded 3 min after measurements commenced to ensure that the recorded signal was stable enough;
- Second, the corrosion rate ($i_{CORR}$) of each sensor was determined by the linear polarisation resistance (LPR) technique. In this method, Stern and Geary’s expression is used to determine $i_{CORR-LPR}$ by estimating polarisation resistance ($R_{p}$) according to Equation (1):
  (1) $$i_{CORR-LPR}=\frac{B}{A\cdot R_{p}}$$
  where A is the surface of the working electrode (1571 mm^2^) and parameter B can adopt values within a range from 13 to 52 mV [80]. In this case, the average value (26 mV) was used, with 2 being the maximum error factor of the prediction [81,82]. Firstly, the corrosion potential ($E_{CORR}$) of the working electrode was recorded when the ∆V/∆t variation reached a value that equaled or was less than 0.03 mV/s. Furthermore, a linear voltammetric scan (LVS) was applied from $E_{CORR}$ − 20 mV to $E_{CORR}$ + 20 mV at a scanning speed of 0.2 mV/s to obtain $R_{p}$ [83,84]. This measurement was taken with an Autolab PGSTAT 100 Potentiostat, and the Nova 1.11 software was used for signal processing. The measurement cell configuration was a 3-electrode one; each sensor was the working electrode. A stainless-steel piece partially immersed in the exposure solution was employed as the counter electrode. The reference electrode was an SCE (Radiometer Analytical XR110), which was also partially immersed in solution.
- Later, the corrosion rate of each sensor was determined by the Potential Step Volmametry (PSV) method ($i_{CORR-PSV}$). This technique, which is used by the INESSCOM system, was introduced in previous works and has been previously validated [71,72,73,74,76]. This measurement was also taken with an Autolab PGSTAT 100 Potentiostat, and the Nova 1.11 software was used for signal processing. The measurement cell configuration was also a 3-electrode one;
- In addition, the sensors’ double-layer capacitance ($C_{DL}$) was determined from the voltammogram (∆I−∆E) obtained after applying CSV, $E_{CORR}$ ± 50 mV × 2 cycles at a sweep speed of 1 mV/s. This procedure, previously used by other authors in the corrosion field [21,22,85], consists of determining the intensity increase corresponding to the voltammeter width in $E_{CORR}$ (${∆I}_{E_{CORR}}$) and replacing it in Equation (2) together with the applied sweep speed (v), which allows to calculate $C_{DL}$.
  (2) $$C_{DL}=\frac{{∆I}_{E_{CORR}}}{2\cdot v}$$

The double-layer capacitance of the steel embedded in concrete is a parameter that has scarcely been valued in studies into the corrosion of RCSs, but its interest as a diagnostic tool is highlighted in this work.

- Finally, the corrosion rate was also determined by the Tafel Extrapolation (TE) method ($i_{CORR-TE}$) as a reference technique [86]. To do this, the polarisation curves ($log\left|∆i\right|$ vs. ∆E) were obtained by applying a linear potential sweep at a sweep speed of 0.2 mV/s [86]. Initially, the sweep was applied in a positive direction from $E_{CORR}$ to $E_{CORR}$ + 140 mV. Subsequently, a 24 h period was used to ensure that $E_{CORR}$ returned to the initially recorded values (with a difference of ±5 mV), and then the scan was applied in a negative direction from $E_{CORR}$ to $E_{CORR}$ − 140 mV [87,88]. Later, $i_{CORR-TE}$ was determined by extrapolating the straight sections (from $E_{CORR}$ ± 59 mV) of the anodic and cathodic curves to $E_{CORR}$ according to [89];
- To complement and compare the information obtained from the sensors by the electrochemical methods described above, a visual inspection of the test specimens was also carried out to visually detect any appreciable corrosion symptoms. To be able to inspect the sensor state, three test specimens from all three groups (A, B, C) were broken once the study was completed.

The statistical analyses of the data obtained from the test specimens were carried out with the R-18 software of R-Project for Statistical Computing. Initially, a univariate analysis of each registered electrochemical parameter ($E_{CORR}$, $C_{DL}$, $i_{CORR-LPR}$, $i_{CORR-PSV}$, $i_{CORR-TE}$) was performed. The average value for each group of samples (A, B, C) was determined, as were the 95% confidence intervals and the corresponding standard deviations. The ANOVA method allowed for the determination of the *p*-value to verify the existence of statistically significant differences among those groups, for which the graphic analysis was not sufficiently enlightening.

A multivariate analysis of the sensors’ responses to the CSV described above was run. This type of scan allows information to be obtained relatively quickly and without significantly altering the sensors’ original $E_{CORR}$. For all 27 sensors, 1312 current density values were recorded. With this raw data, a matrix was defined, where each row corresponds to a sensor (27 sensors) and the columns contain the current density data obtained by CSV (1312 measurements). Therefore, the dimension of the raw data matrix (X) is 27 × 1312, and the sample space will be composed of 1312 variables. These variables correlate with one another, which makes it difficult to analyse them with a univariate approach, and this is why it is necessary to perform a multivariate statistical analysis. The PCA allows the wide set of the original variables to be narrowed to a set of new variables or main components (PCs) that do not correlate with one another. Each PC is a linear combination of the original variables to maximise the variance of the projected data [90]. Therefore, the original matrix X can be decomposed as depicted in Equation (3) [91].

(3) X=S·L+E

The score matrix (S) displays the position of the samples in the new coordinate system. The loadings matrix (L) describes how the new axes have been constructed from the original variables, and E is the matrix of error. As there are only a few samples, the “Leave-One-Out-Cross-Validation” (LOOCV) method was used to calibrate and validate the PCA model. The k-means method was followed to identify the resulting groupings by representing the samples in the new coordinate system. The system was composed of the first two main components that collected the most variance (PC1-PC2). Each sample is considered to belong to the group whose average value is closer [92]. The relation between the resulting groupings and the responses of the sensors in the different environments was analysed.

## 3. Results

Table 2 shows the values obtained by the different techniques described in Section 2.4.

### 3.1. Corrosion Potential and Corrosion Rate

**Figure 3:**
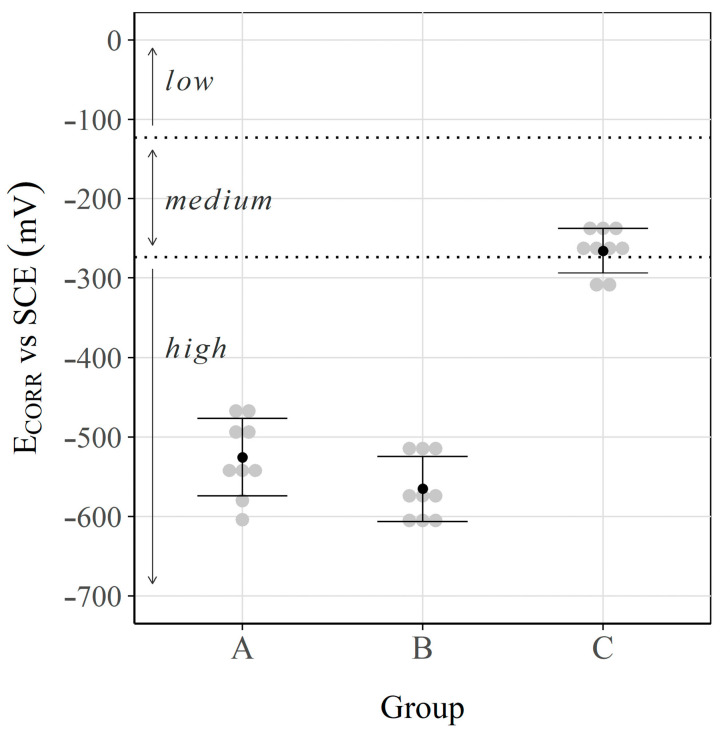
Corrosion potential ($E_{CORR}$) values. Interval plot, which shows the mean and the 95% confidence interval bars of each group.

**Figure 4:**
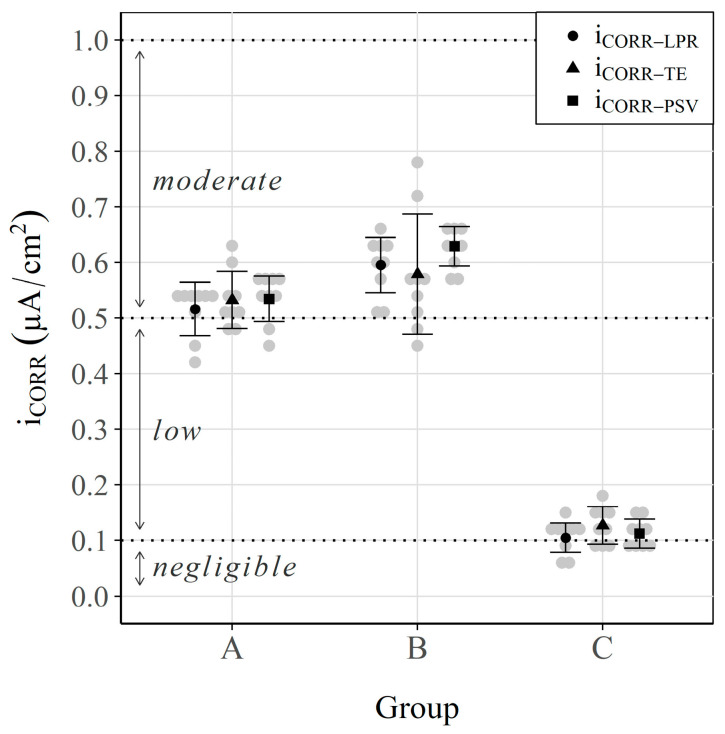
Corrosion rate ($i_{CORR}$) values ($i_{CORR-LPR}$, $i_{CORR-PSV}$ and $i_{CORR-TE}$). Interval plot, which shows the mean and the 95% confidence interval bars of each group.

Figure 3 and Figure 4 show the results obtained for each group: the corrosion potential ($E_{CORR}$) and the corrosion rate ($i_{CORR}$), respectively. The thresholds defined in Standard ASTM-C876 [79] and the RILEM TC 154 EMC recommendation [93] to classify the corrosion risk according to $E_{CORR}$ and the corrosion level according to $i_{CORR}$ are respectively identified in these two figures.

The corrosion potentials of the samples in groups A and B (aggressive environment) went from −450 to −600 mV, which denotes a high risk of undergoing active corrosion processes, in accordance with ASTM-C876 [79]. For the samples in group C (immersed in saturated ${Ca(OH)}_{2}$ solution for 18 months), the values fell between −225 and −300 mV, which denotes a medium-low corrosion risk [79].

**Figure 5:**
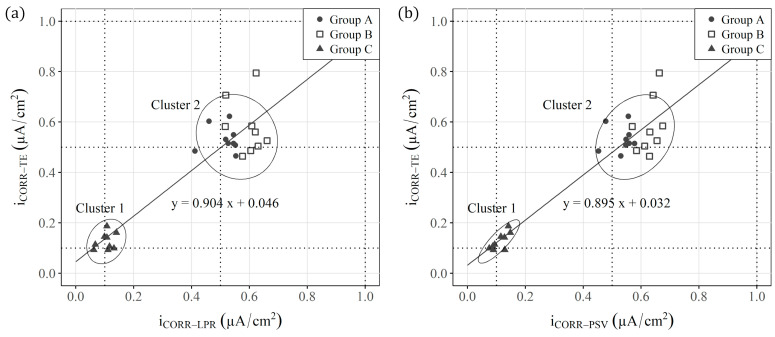
Regression of: (**a**) $i_{CORR-LPR}$ and $i_{CORR-TE}$ and (**b**) $i_{CORR-PSV}$ and $i_{CORR-TE}$.

For the specimens from Group C (non-aggressive environment), values corresponding to negligible corrosion (close to 0.1 μA/cm^2^) were recorded [93]. These data indicate that samples remained under passive conditions. Conversely, with the specimens in groups A and B, which corresponded to aggressive environments, the average values lay between 0.5 and 0.6 μA/cm^2^. According to the RILEM recommendation [93], these values indicate that samples were under moderate corrosion conditions. In addition, Figure 4 shows no significant difference between groups A and B. In fact, when $i_{CORR-LPR}$, $i_{CORR-PSV}$ and $i_{CORR-TE}$ were represented together (Figure 5a,b), only two differentiated groups were observed: one with the samples in a non-aggressive environment (group C) and the other with all the samples in aggressive environments (groups A and B). The value of the slope of the regression line in both figures (Figure 5a,b) demonstrates the deviation that both techniques (LPR and PSV) present compared with the reference Tafel Extrapolation (TE) method. These values (0.904 and 0.895) indicate that the corrosion kinetics are slightly overestimated when calculating the $i_{CORR}$ of both the LPR and PSV techniques in relation to the TE method, with a deviation close to 10%.

### 3.2. Double-Layer Capacitance

**Figure 6:**
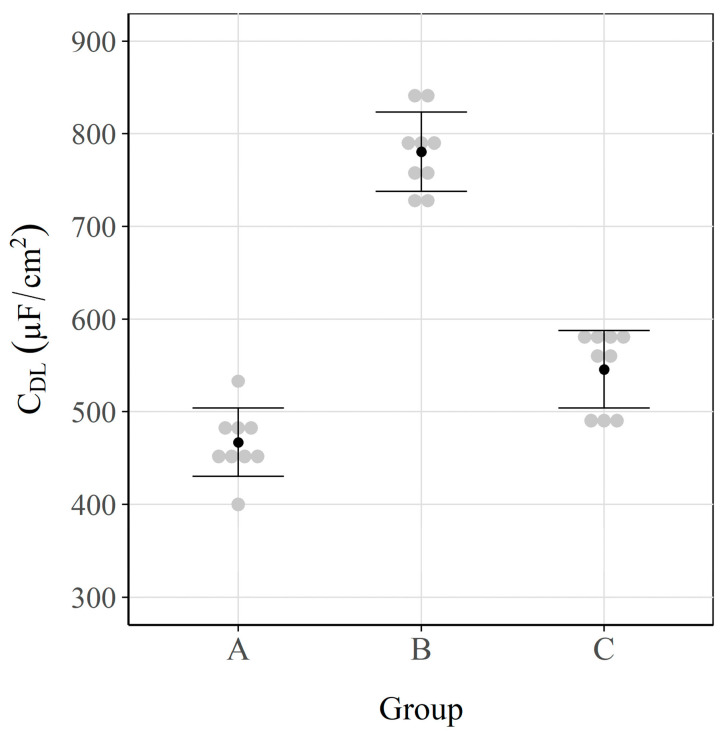
Double-layer capacitance ($C_{DL}$) values. Interval plot, which shows the mean and the 95% confidence interval bars of each group.

Figure 6 shows the $C_{DL}$ average values and their respective 95% confidence intervals for each group of samples.

In this case, there was a considerable difference in the groups of sensors. Of the two groups subjected to aggressive environments (A and B), group B had a higher $C_{DL}$ average (close to 800 μF/cm^2^). This can be explained by the type of agent that triggers the corrosion process. As group B was subjected exclusively to chlorides, corrosion occurred locally in the form of pitting. In Group A (carbonated specimens + chloride exposure), steel corrosion took place uniformly on the surface of the steel. In this case for chloride-induced corrosion, where localised pitting was caused, the microcells that were generated in these zones were supported by the currents that were brought about in the nearby regions still in the passive state (i.e., macrocells), as previous works have revealed [75,94]. Different authors have demonstrated that this produces a pseudocapacitance effect that favours an increase in $C_{DL}$ [95,96]. Conversely, the samples in Group A were firstly carbonated, and although they were later exposed to a solution with chlorides, the corrosion process was uniform over the entire rebar surface. In this case, macrocells were negligible because the whole rebar anodically acted, which favoured the obtained $C_{DL}$ results being lower. These data were validated later by means of visual inspection.

The capacitance values of Group C in the passive state came close to 550 μF/cm^2^. These values were slightly higher than those obtained for Group A (≈470 μF/cm^2^) in the active state (Figure 6). This phenomenon, which has also been observed by other authors [85], comes about because the passive layer that protects metal from corrosion (group C) is formed by compact oxides, which homogeneously cover the entire surface. Conversely, in Group A, there was a similar oxide layer, but it was caused by a uniform corrosion process. To evaluate the differences between groups A and C, which are next to each other in Figure 6, an ANOVA of the $C_{DL}$ results was performed. The obtained *p*-value was 6.3 × 10^−4^, which indicates statistically significant differences between groups A and C.

### 3.3. Visual Inspection

**Figure 7:**
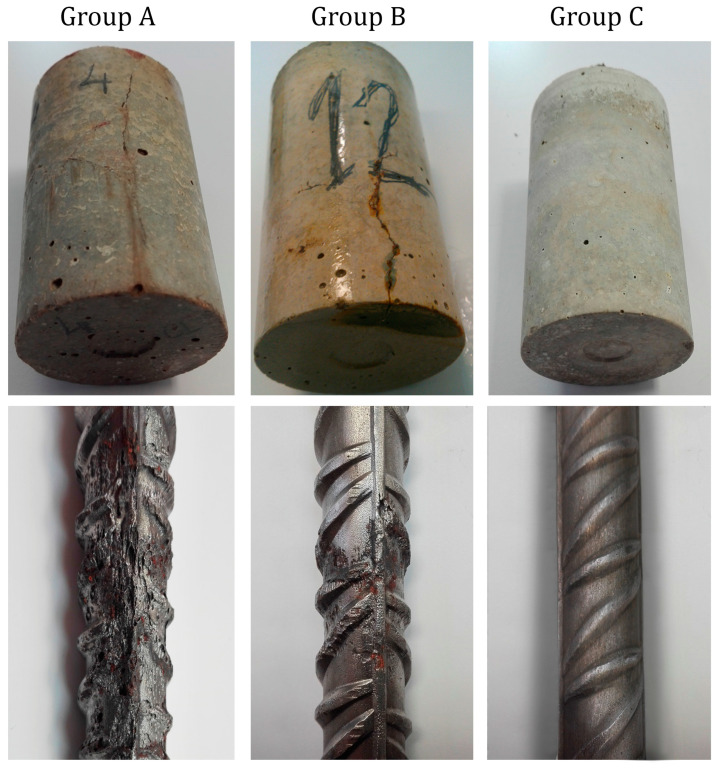
Specimens’ state at the age of 540 days (groups A, B and C).

After taking the different measurements, the test specimens were subjected to visual inspection. No corrosion symptoms were observed in the Group C samples. In groups A and B, cracks and some rust spots were detected on the surfaces of the specimens (Figure 7). To support the $E_{CORR}$ and $i_{CORR}$ results, several samples from each group were broken. The inspection of the surface of the sensors revealed that corrosion in Group A was practically homogeneous, whereas there was pitting in Group B, which caused oxide stains in specific areas (Figure 7).

These data confirmed that the Group C samples remained in the passive state, while those in groups A and B, subjected to aggressive environments, displayed advanced corrosion states.

### 3.4. Statistical Analyses

Chemometric techniques were applied to analyse the information collected by sensors. Multivariate analyses of the experimental data were used to obtain a statistical model capable of clearly differentiating samples based on the dominant corrosion process type (carbonation + chlorides, chlorides, or passivity).

**Figure 8:**
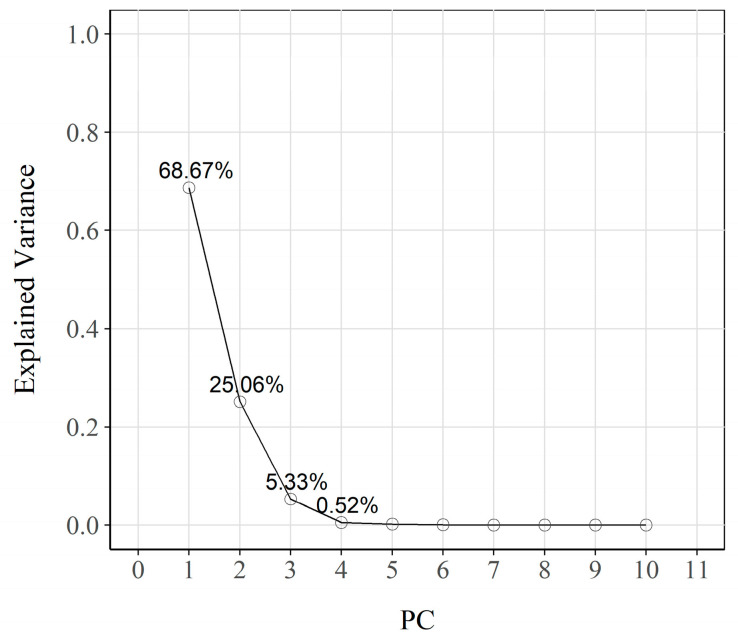
Screen plot of the extracted principal components. The fraction of total variance represented by each principal component is also noted.

For this purpose, the obtained $C_{DL}$ data were used by applying the 2-cycle CSV ($E_{CORR}$ ± 50 mV), as Section 2.4 described. These measurements served to construct a matrix. The analysed matrix consisted of 27 rows (number of sensors) and 1312 columns (one for each current density value recorded every 0.3048 s during the test). After centring and scaling the data matrix, the PCA analysis was performed to generate a new set of uncorrelated variables, or principal components (PCs). The corresponding eigenvalues and their respective variances are shown in the screen plot of Figure 8. Although there is no general criterion for selecting a number of eigenvalues, in this study, the eigenvalues corresponding to the first three PCs were selected because their cumulative variance was over 99%. In addition, the mean square error of the cross-validation (RMSECV) remained practically constant from the third retained component (RMSECV = 1.54), which confirmed the PCA model’s validity. The first main component (PC1) explained 68.67% of the variance, while the second (PC2) accounted for 25.06%. Both PCs together explained a high percentage of the total variance (93.73%).

**Figure 9:**
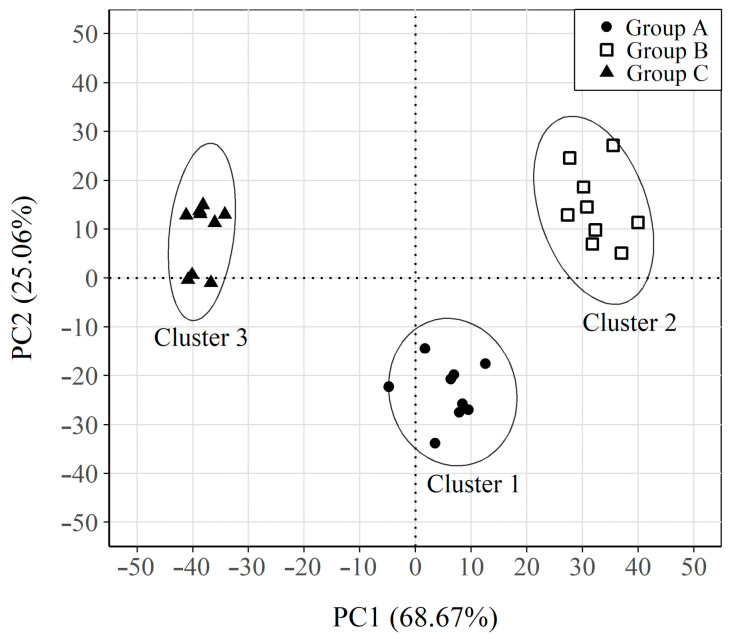
Plot of the samples on the new axes PC1-PC2 system. Samples clustering classification based on K-mean results. Respective 95% confidence ellipses are also displayed.

Figure 9 shows the clustering that resulted from plotting samples in the new PC1-PC2 coordinate system after applying the k-means algorithm. Three clearly differentiated clusters are observed, and each corresponds to a different group of samples (A, B, C).

**Figure 10:**
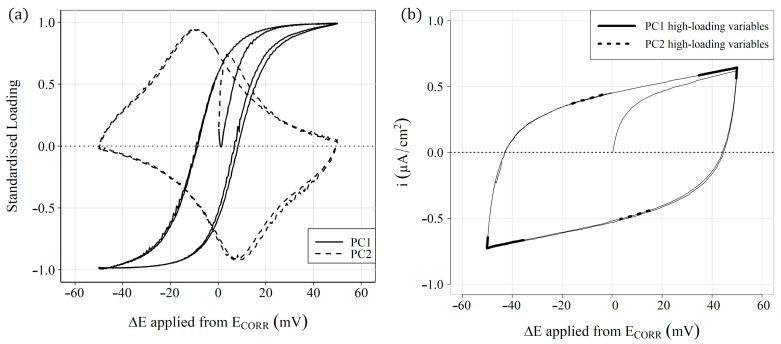
(**a**) Standardised loadings of the original variables for PC1 and PC2 (**b**), the position of which has the highest loadings on one of the polarisation curves (∆I − ∆E).

To understand the nature of the different groups, the contribution of the original variables in PC1 and PC2 is shown in Figure 10a, a graph of the rescaled loadings obtained by multiplying the loadings of each variable in PC1 and PC2 by their corresponding standard deviation. In addition, to facilitate the understanding, the original variables are represented on the *x* axis as the increase in potential (∆E) applied in CSV. The weight of the variables furthest from $E_{CORR}$ (+35/+50 and −35/−50 mV) is considerable in PC1. These variables correspond to the linear sections of the ∆I−∆E curve associated with the faradic current of the oxidation-reduction processes from which the parameter $R_{P}$ was obtained (Figure 10b). The section corresponding to the anodic part of the curve has a positive weight in PC1, while the corresponding cathodic section has a negative weight. Therefore, the samples with positive values in PC1 present high values in the faradic intensity associated with the anodic or cathodic process. These results fall in line with the obtained corrosion rate ($i_{CORR}$) results because the samples with high $i_{CORR}$ values (groups A and B) have positive values in PC1, and those with low $i_{CORR}$ values (Group C) show negative values.

The variables close to $E_{CORR}$ (−16/−4 and +15/+3 mV) are those with the greatest weight in PC2. These variables correspond to the end of the non-linear sections of the ∆I−∆E curve, associated with the charging and discharging processes of the double-layer capacitor ($C_{DL}$) generated at the steel-concrete interface. The corresponding variables of the anodic part of the curve have a positive weight in PC2, while those of cathodic character have a negative weight. Therefore, the results indicate that component PC2 is related mainly to parameter $C_{DL}$ and PC1 is associated with $i_{CORR}$.

**Figure 11:**
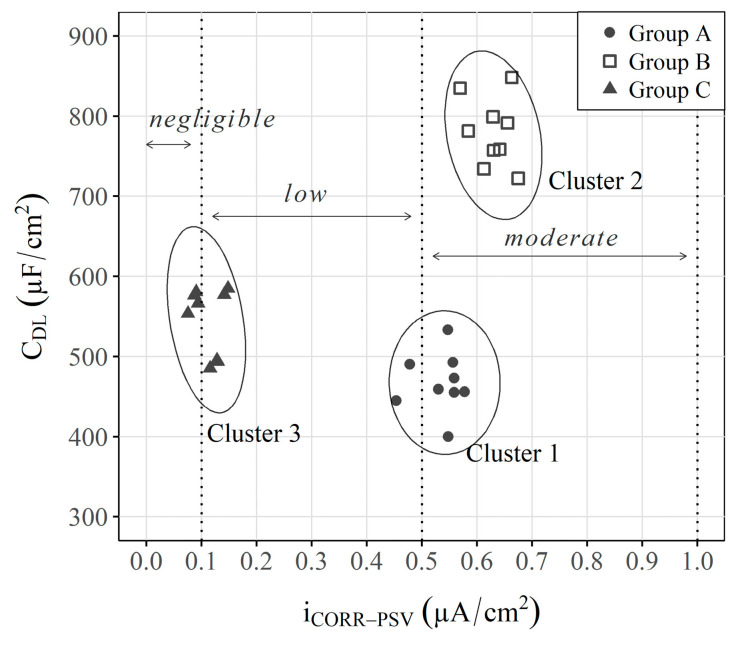
Spontaneous clustering of samples when $i_{CORR-PSV}$ vs. $C_{DL}$ are plotted. Respective 95% confidence ellipse and a hypothetical position of two axes equivalent to the PC1-PC2 axes obtained by PCA (dashed line) are displayed. In addition, the corrosion risk intervals are plotted according to the ranges established for $i_{CORR}$ by Standard UNE 112072:2011 [83].

The PCA is a very useful analysis method because it includes many variables. However, it is difficult to understand because, as indicated, the axes do not refer to a given parameter. To understand this approach, Figure 11 presents a graph that compares the obtained $i_{CORR-PSV}$ and $C_{DL}$ values to offer a simpler interpretation of the data.

The graph in Figure 11 shows a classification that is somewhat similar to that obtained by the PCA, but now the parameters of each axis are known. As depicted in Figure 4 ($i_{CORR-PSV}$) and Figure 5 ($C_{DL}$), the analysis of the independent parameters *per se* does not allow groups to be differentiated. For example, groups A and B have similar corrosion rates, and groups A and C have extremely similar capacitances, which does not allow a suitable classification to be made. On the contrary, when both parameters are represented in a 2-axis system, the three study scenarios are clearly distinguished, but with a more user-friendly representation than that obtained with the PCA.

These data indicate the suitability of implementing and analysing other parameters, like $C_{DL}$ in the INESSCOM monitoring system, because they would help to understand the phenomena that occur internally in RCSs from the rebar corrosion point of view.

## 4. Conclusions

The tests carried out in this research work allow us to draw the following conclusions:

- Obtaining the $E_{CORR}$, $C_{DL}$, and $i_{CORR}$ allows for monitoring the kinetic activity of the embedded sensors in RCSs. Nevertheless, analysing these parameters independently can lead to mistaken interpretations;
- Conversely, the analysis performed by multivariate tools (PCA) of sensors’ ($C_{DL}$ and $i_{CORR}$) responses allows a classification that distinguishes the different study scenarios;
- To facilitate PCA understanding, this work also proposes using comparative graphs of both parameters ($C_{DL}$ and $i_{CORR}$) to distinguish the three study scenarios, but with a much clearer representation in which each axis corresponds to a given parameter, unlike the PCA;
- The obtained results demonstrate that implementing a new measurement protocol in INESSCOM to, in this case, analyse $C_{DL}$, would be extremely useful for simply and quickly determining the precursor corrosion agent, even when the recorded corrosion kinetics are similar.

The results presented in this study were obtained using test specimens under laboratory conditions. In the second part of this study, the double-layer capacitance analysis was also implemented while monitoring the scaled elements exposed to different aggressive environments to analyse the triggering agent of corrosion processes. In this case, the results were also validated by means of visual inspection and a multivariate analysis.

## Figures and Tables

**Table 1:** Concrete mixture proportions (kg/m^3^).

Cement	Water	Sand (0/2)	Sand (2/4)	Gravel (4/6)	w/c Ratio
250	220	735.5	735.5	638	0.8

**Table 2:** Values of the corrosion parameters obtained in the test specimens.

Exposure Condition	Sample	$E_{CORR}$ vs. SCE (mV)	$C_{DL}$ (µF/cm^2^)	$i_{CORR-LPR}$ (µA/cm^2^)	$i_{CORR-PSV}$ (µA/cm^2^)	$i_{CORR-TE}$ (µA/cm^2^)
Group A (Carbonation + Chlorides)	1	−604	473	0.527	0.559	0.515
	2	−470	459	0.553	0.530	0.465
	3	−580	456	0.545	0.578	0.514
	4	−465	490	0.461	0.478	0.603
	5	−538	455	0.546	0.559	0.549
	6	−482	400	0.519	0.548	0.531
	7	−532	492	0.531	0.556	0.622
	8	−552	445	0.412	0.454	0.485
	9	−505	533	0.552	0.548	0.508
	Mean	−525	467	0.52	0.53	0.53
	CoV ^1^	9.0%	7.9%	9.6%	7.7%	9.4%
Group B (Chlorides)	1	−514	835	0.517	0.569	0.582
	2	−515	848	0.623	0.663	0.795
	3	−514	759	0.518	0.641	0.706
	4	−595	781	0.605	0.584	0.487
	5	−607	757	0.620	0.630	0.560
	6	−564	722	0.608	0.674	0.584
	7	−579	734	0.630	0.612	0.505
	8	−585	791	0.661	0.655	0.526
	9	−615	799	0.576	0.629	0.464
	Mean	−565	781	0.59	0.629	0.58
	CoV ^1^	7.3%	5.5%	8.5%	5.6%	18.9%
Group C (Saturated Ca(OH)_2_ solution)	1	−230	554	0.132	0.075	0.099
	2	−240	576	0.116	0.087	0.106
	3	−251	494	0.111	0.123	0.093
	4	−308	567	0.067	0.0937	0.114
	5	−245	580	0.062	0.090	0.093
	6	−309	495	0.107	0.128	0.142
	7	−272	485	0.099	0.115	0.146
	8	−274	577	0.107	0.141	0.187
	9	−258	585	0.140	0.148	0.161
	Mean	−265	546	0.11	0.112	0.13
	CoV ^1^	10.6%	7.7%	27.3%	23.5%	23.1%

^1^ CoV: Coefficient of Variation.

## Data Availability

The data presented in this study are available upon request from the corresponding author.
